# Clinical significance of controlling nutritional status score (CONUT) in evaluating outcome of postoperative patients with gastric cancer

**DOI:** 10.1038/s41598-021-04128-4

**Published:** 2022-01-07

**Authors:** Qi Xiao, Xiaoqing Li, Baojun Duan, Xiaofan Li, Sida Liu, Boyu Xu, Shuai Shi, Jin Zhang, Haoyuan Qin, Xianglong Duan, Yansong Pu

**Affiliations:** 1 Second Department of General Surgery, Shaanxi Provincial People’s Hospital, Xi’an, 710068 Shannxi Province China; 2 Department of Dermatology, Shaanxi Provincial People’s Hospital, Xi’an, 710068 Shannxi Province China; 3 Department of Oncology, Shaanxi Provincial People’s Hospital, Xi’an, 710068 Shannxi Province China; 4 Xi’an Medical University, Xi’an, 710068 Shannxi Province China; 5 Yan’an University, Yan’an, 716000 Shannxi Province China; 6 Second Department of General Surgery, Affiliated Hospital of Northwestern Polytechnical University, Xi’an, 710068 Shannxi Province China

**Keywords:** Gastric cancer, Malnutrition

## Abstract

The stomach is the main digestive organ in humans. Patients with gastric cancer often develop digestive problems, which result in poor nutrition. Nutritional status is closely related to postoperative complications and quality of life (QoL) in patients with gastric cancer. The controlling nutritional status (CONUT) score is a novel tool to evaluate the nutritional status of patients. However, the relationship of the CONUT score with postoperative complications, QoL, and psychological status in patients with gastric cancer has not been investigated. The present follow-up study was conducted in 106 patients who underwent radical gastrectomy in our hospital between 2014 and 2019. The CONUT score, postoperative complications, psychological status, postoperative QoL scores, and overall survival (OS) of patients with gastric cancer were collected, and the relationship between them was analyzed. A significant correlation was observed between the CONUT score and postoperative complications of gastric cancer (*P* < 0.001), especially anastomotic leakage (*P* = 0.037). The multivariate regression analysis exhibited that the CONUT score (*P* = 0.002) is an independent risk factor for postoperative complications. The CONUT score was correlated with the state anxiety questionnaire (S-AI) for evaluating psychological status (*P* = 0.032). However, further regression analysis exhibited that the CONUT score was not an independent risk factor for psychological status. Additionally, the CONUT score was associated with postoperative QoL. The multivariate regression analysis exhibited that the CONUT score was an independent risk factor for the global QoL (*P* = 0.048). Moreover, the efficiency of CONUT score, prognostic nutrition index, and serum albumin in evaluating complications, psychological status, and QoL was compared, and CONUT score was found to outperform the other measures (Area Under Curve, AUC = 0.7368). Furthermore, patients with high CONUT scores exhibited shorter OS than patients with low CONUT scores (*P* = 0.005). Additionally, the postoperative complications (HR 0.43, 95% CI 0.21–0.92, *P* = 0.028), pathological stage (HR 2.26, 95% CI 1.26–4.06, *P* = 0.006), and global QoL (HR 15.24, 95% CI 3.22–72.06, *P* = 0.001) were associated with OS. The CONUT score can be used to assess the nutritional status of patients undergoing gastric cancer surgery and is associated with the incidence of postoperative complications and QoL.

## Introduction

Gastric cancer has become a common malignant disease, with high incidence and mortality in modern China because of the accelerated pace of life and dietary changes^1^. Approximately 0.48 million new cases of gastric cancer are reported annually in China. The mortality rate of gastric cancer is second only to lung cancer and colorectal cancer^2^.

The stomach is the main digestive organ of the human body^3^. Patients with gastric cancer often experience indigestion symptoms such as stomachache, abdominal distension, nausea, and vomiting, which affect food intake. Therefore, patients with gastric cancer are more likely to have malnutrition than patients with other malignant tumors^4^. Perioperative malnutrition may increase the incidence of postoperative complications and affect the quality of life (QoL), psychological status, and OS of patients. The preoperative nutritional status of patients must be accurately evaluated and the appropriate nutritional intervention be administered to patients with poor nutritional status to minimize the incidence of postoperative complications and improve the survival rate and QoL of patients with gastric cancer^5–7^. Malnutrition and malignant tumors form a vicious circle where one promotes the other.

An increasing number of clinicians have realized the significance of nutrition in patients with gastric cancer. Therefore, the nutritional status of patients with gastric cancer must be accurately assessed. Nutrition evaluation systems such as NRS2002 and patient-generated subjective global assessment (PG-SGA) are widely used in the clinic for patients with tumors. However, these assessment methods have significant drawbacks due to the tedious, complex, time-consuming, and subjective processes. First, it is limited by the memory and knowledge level of patients; several patients cannot recall and provide the nutritional status accurately. Second, the subjective evaluation of doctors affects the accuracy of conclusions. PG-SGA is associated with similar limitations. Moreover, traditional nutritional assessment tools are subjective and complex, making it difficult for general clinicians to accurately evaluate the nutritional status of patients and efficacy of nutritional interventions. Therefore, the present study attempted to establish a simple and objective evaluation method.

The controlling nutritional status (CONUT) score is an objective nutrition evaluation index based on serum albumin (ALB), total cholesterol, and lymphocyte count. The nutrition score of patients can be easily obtained by delineating the score range of these indices and has a superior evaluation effect on the nutritional status of patients with cancers^8^. The CONUT score ranges between 0 and 9, and it more accurately reflects the effect of nutritional treatment than other evaluation systems. It is helpful in the perioperative patient management, diagnosis, and treatment planning. The laboratory results of this score are easy to obtain, the evaluation process is simple, and the clinical use of this score is obviously more convenient than that of other tools. The CONUT score was shown to be critical in the nutritional evaluation of patients with gastrointestinal or pulmonary tumors^9^. However, limited evidence is available to confirm its accuracy. Therefore, the present retrospective study attempted to clarify whether the CONUT score could accurately assess the preoperative nutritional status of patients with gastric cancer and its association with postoperative complications, QoL, and psychological status.

## Patients and methods

### Statements

The use of clinical data and other follow up procedure in this study were approved by the Human Ethics Review Committee of Shaanxi Provincial People's Hospital (MEC code: SPPH-LLBG-17-3.2).

All participants gave informed consent to the research, allowed access to their clinical data, agreed to receive follow-up and questionnaire surveys, and signed an informed consent form which based on CIOMS guidelines.

### Patients

The present study extracted the data of 168 patients, aged more than 18 years, with gastric cancer who underwent gastric cancer-related surgery for the first time from 2014 to 2019 in Shaanxi Provincial People's Hospital. Patients with incomplete data and those who could not be followed up were excluded from the study. Finally, the data of 106 patients were collected to study the correlation of the preoperative CONUT score with postoperative complications, psychological status, and QoL of patients with gastric cancer. The state–trait anxiety inventory (STAI) was used to measure the psychological state and anxiety level of patients. EORTC QLQ-C30 (version 3) was used to evaluate the postoperative QoL of patients.

Data were extracted from medical records, and telephone and web follow-ups were also conducted. The median follow-up period was 30 months (range 7–64 months).

## Methods

All methods in this study were carried out in accordance with relevant guidelines and regulations of Shaanxi Provincial People's Hospital.

### CONUT score, complications, and other scoring systems

According to the CONUT score items, data on the lymphocyte count, ALB, and total cholesterol levels of patients with gastric cancer were collected within 2 weeks before the surgical procedure, whereas the data on carcinoembryonic antigen (CEA) and carbohydrate antigen 19-9 (CA19-9) levels were collected within 1 month before the operation. The CONUT score was calculated, and all the patients were divided into low CONUT (CONUT < 5) and high CONUT groups (CONUT ≥ 5), with a median score of 5 (range 3–9).

Surgical methods included both laparoscopy and open surgery. The resection range included procedures such as total gastrectomy, subtotal gastrectomy, and distal gastrectomy. Common complications included pulmonary infection, bleeding, anastomotic leakage, anastomotic stenosis, organ failure, severe systemic infection, surgical incision or anastomotic infection, and vascular events (thrombosis). Simultaneously, the classification of surgical complications was determined according to the Clavien–Dindo classification^10^. We only sorted out the data of the grades II–V complications as the complications of grade I were mild and had little impact on the prognosis.

The postoperative psychological status of the patients was assessed using the STAI (STAI-Formy, S-AI/T-AI). STAI comprises the following two subscales: state anxiety questionnaire (S-AI) and trait anxiety questionnaire (T-AI), each with 20 items. Half of the S-AI (items 1–20) are items describing negative emotions, whereas the other half are items describing positive emotions. These items can be used to evaluate state anxiety under stress. In T-AI (items 21–40), 11 items are negative emotion items, whereas 9 items are positive emotion items. These items are used to assess the frequent and perennial emotional experiences of people.

The EORTC QLQ-C30 (version 3) was used to evaluate the postoperative QoL. The QLQ-C30 contains 30 items, which can be divided into 15 areas, including 5 functional areas (physical function, role function, emotional function, cognitive function, and social function), 3 symptom areas (fatigue, pain, nausea, and vomiting), 1 global health status or QoL area, and 6 single items. High scores in the functional and overall health status fields indicate better functional status and life quality, whereas high scores in the symptom field indicate more symptoms or problems (a lower life quality). Therefore, the raw QLQ-C30 scores were transformed into standard scores for further analysis.

Because of the randomness of remote follow-up and the uncertainty of psychological investigation, a series of strict standards were formulated to minimize the interference factors in the follow-up process under the existing conditions. The content of follow-up was determined in advance, standardized instructions were formulated, and staff were designated in a quiet environment to follow-up from 9 a.m. to 11 a.m., 3–6 p.m., and 7 p.m. to 9 p.m. Each patient was explained the purpose and content of the follow-up and the time required for the same. The study was initiated only after the patient informed consent for the same. We helped the patients recall the psychological changes before operation and the postoperative influence. Several remedial strategies were developed to avoid communication errors and ensure the efficiency of follow-up.

**Figure 1:**
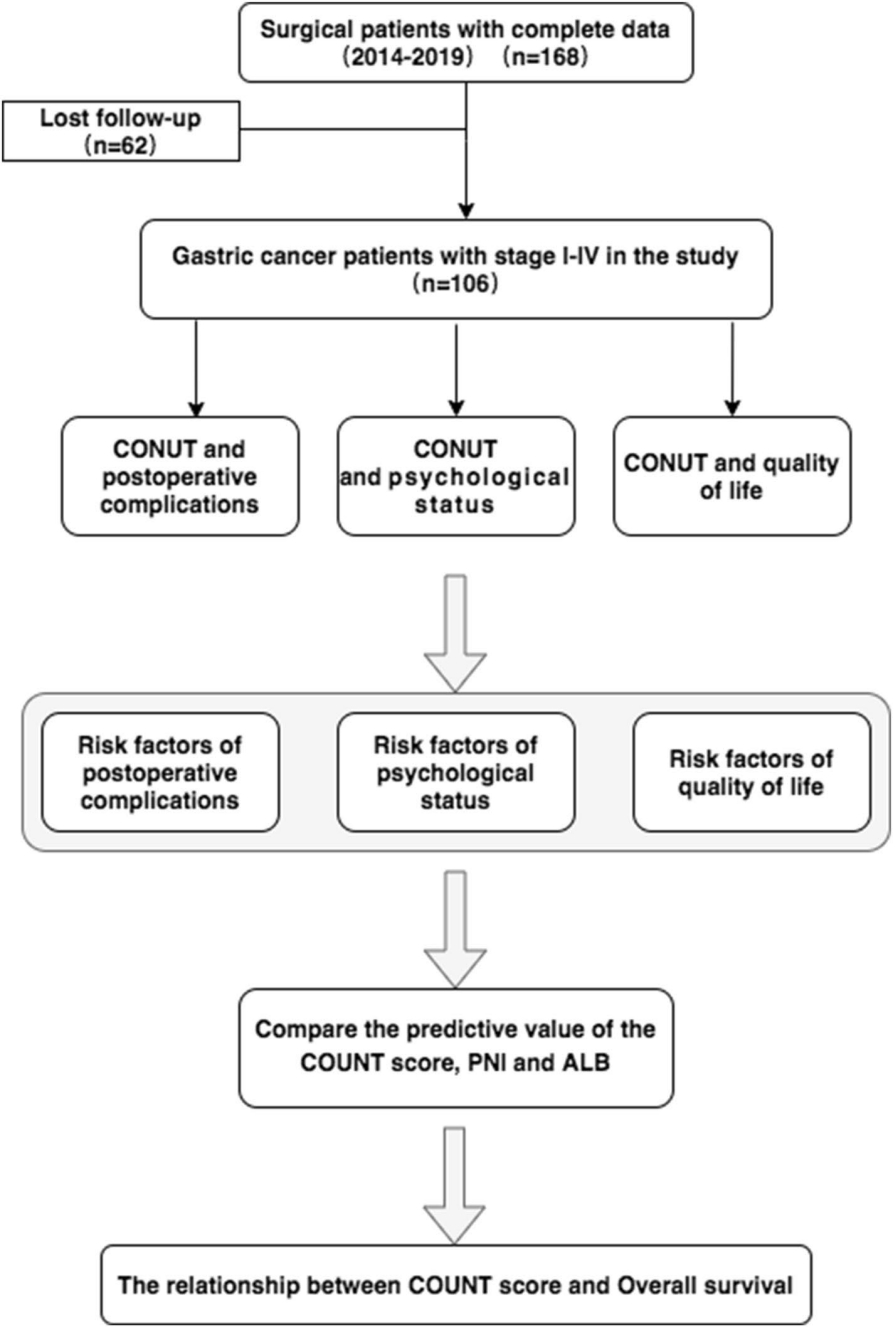
Flow chart of utilizing the value of CONUT score in the study of complications, psychological status, and QoL in gastric cancer patients.

To better assess the predictive value of the CONUT score, two classical indicators of nutritional assessment, namely the prognostic nutritional index (PNI) and ALB content, were introduced for comparison. The experimental flow is illustrated in Fig. 1.

### Statistical analysis

All data were analyzed using Excel 2019 (Microsoft, Redmond, WA, USA) and the SPSS Statistics software, versions 22.0 and 26.0 (IBM Corporation, Armonk, NY, USA). *P* value < 0.05 was considered statistically significant.

Continuous variables are expressed as median (quartile range) or mean ± standard deviation. Classification variables are represented as numbers (%). The t test was used to assess differences in continuous variables such as patient age, length of stay, CA19-9, and STAI. Chi-square test or Fisher's exact test was used to analyze and compare categorical variables such as sex, tumor stage, and complications. Logistics regression analysis was used to analyze whether the CONUT score is an independent risk factor for complications, STAI, and EORTC QLQ-C30. COX regression was used to analyze the relationship between CONUT score and OS. An ROC curve was used to compare the predictive values of CONUT, PNI, and ALB.

## Results

### Patient characteristics

The median age of the patients was 67 years (43–85 years). According to the World Health Organization age classification criteria, the patients were divided into two groups, namely < 60 years old and ≥ 60 years old. A total of 44 patients (41.5%) died by the time of follow-up. Finally, the study group comprised 84 men (79.2%) and 22 women (20.8%). Of the 106 patients, 17 patients (16%) were in pathology stage I, 20 patients (18.9%) were in stage II, 57 patients (53.8%) were in stage III, and 12 patients (11.3%) were in stage IV as per the tumor, nodes, and metastases (TNM) staging criteria of the National Comprehensive Cancer Network. The chi-square test indicated no correlation between the CONUT score and TNM stage. However, sex (*P* = 0.016), survival time (*P* = 0.005), CA19-9 (*P* = 0.021), and surgical resection range (*P* = 0.024) were found to be significantly correlated with the CONUT score. Postoperative complications were observed in 39 patients (36.8%). A significant association was observed between CONUT score and postoperative complications (*P* < 0.001) (Table 1).

**Table 1:** Patient characteristics (n = 106).

	Total (n = 106)	CONUT score		*P* value
		< 5 (n = 43)	≥ 5 (n = 63)	
**Age, years**				0.600
< 60	22 (20.8%)	10 (23.3%)	12 (19.0%)	
≥ 60	84 (79.2%)	33 (76.7%)	51 (81.0%)	
**Sex**				**0.016**
Male	84 (79.2%)	39 (90.7%)	45 (71.4%)	
Female	22 (20.8%)	4 (9.3%)	18 (28.6%)	
**Survival state**				0.458
Death	44 (41.5%)	16 (37.2%)	28 (44.4%)	
Life	62 (58.5%)	27 (62.8%)	35 (55.6%)	
**Stage**				0.323
I	17 (16.0%)	10 (23.3%)	7 (11.1%)	
II	20 (18.9%)	9 (20.9%)	11 (17.5%)	
III	57 (53.8%)	20 (46.5%)	37 (58.7%)	
IV	12 (11.3%)	4 (9.3%)	8 (12.7%)	
**Complications**				**< 0.001**
Y	39 (36.8%)	6 (14.0%)	33 (52.4%)	
N	67 (63.2%)	37 (86.0%)	30 (47.6%)	
**Survival time, months**		25.84 ± 15.09	17.6 ± 14.36	**0.005**
**Hospital stay, days**		24.28 ± 7.35	25.56 ± 7.45	0.386
**CEA**		39.02 ± 164.36	4.90 ± 9.22	0.181
**CA19-9**		8.93 ± 6.55	39.33 ± 100.77	**0.021**
**Surgical methods**				0.214
Laparoscopy	30 (28.3%)	15 (34.9%)	15 (23.8%)	
Open	76 (71.7%)	28 (65.1%)	48 (76.2%)	
**Resection**				**0.024**
Whole stomach	60 (56.6%)	30 (69.8%)	30 (47.6%)	
Others	46 (21.7%)	13 (30.2%)	33 (52.4%)	

### Correlation between CONUT score and postoperative complications

Gastric cancer exhibits several postoperative complications such as pulmonary infection, bleeding, anastomotic leakage, and anastomotic stenosis. A significant correlation was observed between anastomotic leakage and CONUT score (*P* = 0.037). The severity of complications can be divided into the following 5 grades: Grade I: does not require medical and surgical treatment; Grade II: requires medication, blood transfusion, or total parenteral nutrition; Grade III: requires surgery and endoscopy; Grade IV: exhibits life-threatening complications; Grade V: patient dies. The relationship between the complication grade and CONUT score was further analyzed. Grade II complications were found to be significantly correlated with the CONUT score (*P* = 0.004) (Table 2).

**Table 2:** Correlation analysis of various complications and the CONUT score.

	Total (n = 106)	CONUT score		*P* value
		< 5 (n = 43)	≥ 5 (n = 63)	
Pulmonary infection	12 (11.3%)	2 (4.7%)	10 (15.9%)	0.073
Hemorrhage	6 (5.7%)	1 (2.3%)	5 (7.9%)	0.22
Anastomotic leakage	6 (5.7%)	0 (0.0%)	6 (9.5%)	**0.037**
Anastomotic stenosis	4 (3.8%)	0 (0.0%)	4 (6.3%)	0.092
Organ failure	3 (2.8%)	1 (2.3%)	2 (3.2%)	0.796
Severe sepsis	2 (1.9%)	0 (0.0%)	2 (3.2%)	0.238
Incision infection	3 (2.8%)	1 (2.3%)	2 (3.2%)	0.796
Vascular events	3 (2.8%)	1 (2.3%)	2 (3.2%)	0.796
Complication grade II	22 (20.8%)	3 (7.1%)	19 (30.2%)	**0.004**
Complication grade III	4 (3.8%)	0 (0.0%)	4 (6.3%)	0.092
Complication grade IV	8 (7.5%)	2 (4.7%)	6 (9.5%)	0.351
Complication grade V	5 (4.7%)	1 (2.3%)	4 (6.3%)	0.337

Stratified analysis based on tumor stage showed that patients with high CONUT scores in TNM stage I patients were prone to postoperative complications of grade II (*P* = 0.023) (Table 3).

**Table 3:** The relationship between complications and CONUT score in the hierarchical analysis of tumor stage.

	Total (n = 106)	CONUT score		*P* value
		< 5 (n = 43)	≥ 5 (n = 63)	
**Stage I**				
Anastomotic leakage	2 (1.9%)	0 (0.0%)	2 (3.2%)	0.072
Complication grade II	3 (2.83%)	0 (0.0%)	3 (4.8%)	**0.023**
**Stage II**				
Anastomotic leakage	0 (0.0%)			
Complication grade II	2 (1.9%)	0 (0.0%)	2 (3.2%)	0.178
**Stage III**				
Anastomotic leakage	3 (2.8%)	0 (0.0%)	3 (4.8%)	0.191
Complication grade II	14 (13.2%)	2 (4.7%)	12 (19.0%)	0.06
**Stage IV**				
Anastomotic leakage	1 (0.9%)	0 (0.0%)	1 (1.6%)	0.46
Complication grade II	3 (2.8%)	1 (2.3%)	2 (3.2%)	> 0.05

### Risk factors for postoperative complications

The CONUT score (*P* < 0.001), PNI (*P* = 0.05), ALB (*P* = 0.004), and pathological stage (*P* = 0.004) were found to be significantly correlated with postoperative complications (Table 4).

**Table 4:** Analysis of risk factors of postoperative complications.

	Total (n = 106)	Complications		*P* value
		Y (n = 39)	N (n = 67)	
**Age, years**				0.298
< 60	22 (20.8%)	6 (15.4%)	16 (23.9%)	
≥ 60	84 (79.2%)	33 (84.6%)	51 (76.1%)	
**Sex**				0.344
Male	84 (79.2%)	29 (74.4%)	55 (82.1%)	
Female	22 (20.8%)	10 (25.6%)	12 (17.9%)	
**CONUT**				**< 0.001**
< 5	43 (40.6%)	6 (15.4%)	37 (55.2%)	
≥ 5	63 (59.4%)	33 (84.6%)	30 (44.8%)	
**PNI**				**0.05**
≤ 43.1	52 (49.1%)	24 (61.5%)	28 (41.8%)	
> 43.1	54 (50.9%)	15 (38.5%)	39 (58.2%)	
**ALB**				**0.004**
≤ 35	36 (34.0%)	20 (51.3%)	16 (23.9%)	
> 35	70 (66.0%)	19 (48.7%)	51 (76.1%)	
**Stage**				**0.004**
I	17 (16.0%)	4 (10.3%)	13 (19.4%)	
II	20 (18.9%)	2 (5.1%)	18 (26.9%)	
III	57 (53.8%)	25 (64.1%)	32 (47.8%)	
IV	12 (11.3%)	8 (20.5%)	4 (6.0%)	
**Surgical methods**				0.071
Laparoscopy	30 (28.3%)	7 (17.9%)	23 (34.3%)	
Open	76 (71.7%)	32 (82.1%)	44 (65.7%)	
**Resection**				0.662
Whole stomach	60 (56.6%)	21 (53.8%)	39 (58.2%)	
Others	46 (43.4%)	18 (46.2%)	28 (41.8%)	
**CEA**		44.78 ± 174.48	4.16 ± 6.73	0.16
**CA19-9**		31.00 ± 71.17	24.54 ± 83.15	0.688

### Independent risk factors of complications

To further analyze the independent risk factors for postoperative complications, we performed univariate and multivariate analyses of the CONUT score, PNI, and other indicators which affected complications. The CONUT score (HR 0.15, 95% CI 0.06–0.55, *P* = 0.002) was demonstrated to be an independent risk factor for postoperative complications (Table 5).

**Table 5:** Univariate and multivariate analyses of complications.

	Univariate analysis			Multivariate analysis		
	HR	95% CI	*P* value	HR	95% CI	*P* value
Age, < 60 versus ≥ 60	1.73	0.61–4.86	0.302			
Sex	0.63	0.24–1.64	0.346			
p Stage, II III IV versus I	2.11	0.64–1.98	0.223			
CONUT, low versus high	0.15	0.06–0.40	**< 0.001**	0.15	0.06–0.55	**0.002**
PNI, low versus high	2.23	0.99–5.00	0.052			
ALB, low versus high	3.36	1.45–7.79	**0.005**	1.58	0.61–4.14	0.348
Surgical methods, laparoscopy versus open	0.42	0.16–1.09	0.076			
Resection, whole versus others	1.19	0.54–2.64	0.662			
CA19-9	1.77	0.80–3.94	0.16			

### Relationship of CONUT score with psychological status

Nutritional status has been reported to be related to postoperative psychological status. Therefore, STAI was used to evaluate postoperative psychological status, and the relationship between CONUT score and STAI was analyzed. The S-AI score, which reflects the anxiety in the postoperative stress state, was found to be significantly correlated with the CONUT score (*P* = 0.032) (Table 6). However, the T-AI score, which reflects emotional experiences, demonstrated no obvious correlation with the CONUT score (*P* = 0.058).

**Table 6:** Analysis of the significant difference between CONUT score and patient psychology status.

	CONUT score		*P* value
	< 5 (n = 43)	≥ 5 (n = 63)	
S-AI	35.6 ± 3.02	37.13 ± 4.16	**0.032**
T-AI	37.86 ± 3.02	39.27 ± 4.13	0.058

The results of stratified analysis based on TNM staging showed that stage III patients with high CONUT scores had higher S-AI scores (*P* = 0.02) (Table 7). It was suggested that stage III patients with high CONUT scores were more prone to anxiety.

**Table 7:** The relationship between STAI and CONUT scores in the hierarchical analysis of tumor stage.

	CONUT score		*P* value
	< 5 (n = 43)	≥ 5 (n = 63)	
**Stage I**			
S-AI	35.4 ± 2.37	35.7 ± 4.72	0.875
T-AI	37.4 ± 3.95	41.1 ± 3.89	0.072
**Stage II**			
S-AI	36.4 ± 4.25	36.3 ± 3.98	0.927
T-AI	38.9 ± 3.59	38.2 ± 5.06	0.729
**Stage III**			
S-AI	35.2 ± 2.74	37.4 ± 4.34	**0.02**
T-AI	37.7 ± 2.13	39.1 ± 4.12	0.101
**Stage IV**			
S-AI	36.5 ± 3.42	38.3 ± 3.15	0.397
T-AI	37.5 ± 3.70	40.0 ± 2.88	0.224

### Independent STAI risk factors

Logistic regression analysis was conducted to determine whether the CONUT score is an independent risk factor for the STAI score. However, the CONUT score was not found to be an independent risk factor for both the S-AI (Table 8) and T-AI scores (Table 9).

**Table 8:** Logistic regression analysis of independent risk factors for S-AI.

	Univariate analysis		
	HR	95% CI	*P* value
Age, < 60 versus ≥ 60	0.92	0.36–2.40	0.869
Sex	1.47	0.57–3.77	0.423
p Stage, II III IV versus I	1.66	0.58–4.70	0.343
CONUT, low versus high	0.76	0.35–1.66	0.485
PNI, low versus high	1.01	0.47–2.19	0.976
ALB, low versus high	0.75	0.33–1.68	0.477
Surgical methods, laparoscopy versus open	1.15	0.49–2.72	0.748
Resection, whole versus others	0.79	0.37–1.73	0.56
CA19-9	1.26	0.58–2.73	0.556

**Table 9:** Logistic regression analysis of independent risk factors for T-AI.

	Univariate analysis		
	HR	95% CI	*P* value
Age, < 60 versus ≥ 60	1.17	0.45–3.00	0.749
Sex	0.92	0.36–2.40	0.869
p Stage, II III IV versus I	0.94	0.33–2.70	0.908
CONUT, low versus high	0.47	0.21–1.03	0.059
PNI, low versus high	1.89	0.86–4.13	0.111
ALB, low versus high	1.25	0.55–2.83	0.595
Surgical methods, laparoscopy versus open	0.45	0.19–1.05	0.066
Resection, whole versus others	1.49	0.68–3.27	0.317
CA19-9	2.02	0.92–4.42	0.079

### Relationship of CONUT score with QoL

The nutritional status has been reported to be associated with postoperative QoL. Therefore, EORTC QLQ-C30 was used to evaluate postoperative QoL. Most of the postoperative QoL assessment sub-items were found to be significantly correlated with the CONUT score (Table 10).

**Table 10:** Analysis of the relationship between CONUT and postoperative QoL.

	CONUT score		*P* value
	< 5 (n = 43)	≥ 5 (n = 63)	
Physical function	78.29 ± 14.70	66.67 ± 21.05	**0.001**
Role function	87.98 ± 21.62	73.81 ± 27.71	**0.004**
Emotional function	85.01 ± 16.72	74.34 ± 20.48	**0.004**
Cognitive function	84.88 ± 16.99	73.28 ± 18.58	**0.001**
Social function	84.11 ± 21.50	71.43 ± 24.76	**0.007**
Global QoL	65.70 ± 19.26	51.19 ± 25.35	**0.001**
Fatigue	36.18 ± 20.15	48.15 ± 28.71	**0.013**
Nausea and vomit	4.26 ± 14.59	3.96 ± 9.32	0.899
Pain	7.36 ± 15.98	21.96 ± 29.91	**0.002**
Dyspnea	11.63 ± 17.64	23.81 ± 28.35	**0.008**
Sleep disturbance	30.23 ± 28.93	36.51 ± 28.53	0.271
Appetite loss	17.83 ± 22.24	26.46 ± 28.81	0.101
Constipation	0.78 ± 5.08	3.70 ± 18.07	0.227
Diarrhea	13.18 ± 22.00	10.05 ± 19.52	0.444
Financial difficulties	11.63 ± 20.42	22.22 ± 24.68	**0.022**

In addition, we analyzed the relationship between CONUT scores and preoperative QoL. The CONUT score and preoperative QoL in "Nausea and Vomit (*P* < 0.001), Appetite loss (*P* < 0.001), and Diarrhea (*P* = 0.002)" had significant relationship (Table 11).

**Table 11:** The relationship between CONUT and preoperative QoL.

	CONUT score		*P* value
	< 5 (n = 43)	≥ 5 (n = 63)	
Physical function	58.0 ± 17.73	51.9 ± 15.21	0.06
Role function	57.8 ± 17.95	52.6 ± 22.64	0.219
Emotional function	48.1 ± 15.63	49.2 ± 13.94	0.694
Cognitive function	44.2 ± 18.86	41.3 ± 12.65	0.378
Social function	45.7 ± 15.47	44.7 ± 21.76	0.777
Global QoL	35.7 ± 11.55	40.6 ± 14.24	0.061
Fatigue	53.7 ± 13.70	54.3 ± 16.83	0.853
Nausea and vomit	4.3 ± 14.59	52.1 ± 25.13	**< 0.001**
Pain	54.7 ± 17.94	55.0 ± 18.36	0.917
Dyspnea	53.5 ± 19.44	58.2 ± 28.69	0.316
Sleep disturbance	47.3 ± 29.31	52.9 ± 30.31	0.344
Appetite loss	14.7 ± 19.66	49.2 ± 35.35	**< 0.001**
Constipation	58.9 ± 21.62	64.6 ± 23.85	0.218
Diarrhea	13.2 ± 21.99	29.1 ± 29.63	**0.002**
Financial difficulties	61.2 ± 24.05	60.8 ± 22.03	0.931

The results of the stratified analysis based on tumor stage showed that the postoperative emotional function (*P* = 0.0225), cognitive function (*P* = 0.025), global QoL (*P* = 0.044) and dyspnea (*P* = 0.03) of patients in stage III were significantly correlated with the CONUT score (Table 12).

**Table 12:** The relationship between postoperative QoL and CONUT score in the hierarchical analysis of tumor stage.

	CONUT score		*P* value
	< 5 (n = 43)	≥ 5 (n = 63)	
**Stage I**			
Physical function	85.3 ± 6.13	81.9 ± 13.18	0.479
Role function	96.7 ± 10.54	88.1 ± 15.85	0.199
Emotional function	87.5 ± 10.58	77.4 ± 23.43	0.244
Cognitive function	90.0 ± 14.05	83.3 ± 13.61	0.345
Social function	90.0 ± 14.05	85.7 ± 17.82	0.587
Global QoL	77.5 ± 7.90	71.4 ± 17.25	0.411
Fatigue	25.6 ± 15.76	38.1 ± 23.0	0.2
Pain	1.67 ± 5.27	21.4 ± 39.34	0.234
Dyspnea	10.0 ± 16.1	0	0.081
Financial difficulties	3.3 ± 10.54	9.5 ± 16.26	0.354
**Stage II**			
Physical function	83.0 ± 10.06	72.1 ± 19.28	0.126
Role function	96.3 ± 11.11	78.8 ± 26.97	0.071
Emotional function	82.4 ± 28.09	73.5 ± 20.01	0.418
Cognitive function	87.0 ± 18.21	75.8 ± 15.57	0.153
Social function	88.9 ± 23.57	75.8 ± 21.56	0.21
Global QoL	67.6 ± 18.84	59.1 ± 25.94	0.423
Fatigue	37.0 ± 22.91	46.5 ± 28.47	0.433
Pain	3.7 ± 11.11	18.2 ± 29.30	0.154
Dyspnea	7.4 ± 14.70	12.1 ± 30.81	0.679
Financial difficulties	11.1 ± 23.57	21.2 ± 34.23	0.463
**Stage III**			
Physical function	74.7 ± 17.11	65.8 ± 20.56	0.105
Role function	83.3 ± 27.57	75.2 ± 25.34	0.268
Emotional function	86.7 ± 11.91	76.6 ± 21.05	**0.025**
Cognitive function	84.2 ± 14.78	73.4 ± 17.77	**0.025**
Social function	80.8 ± 21.81	70.7 ± 23.37	0.117
Global QoL	62.1 ± 21.20	49.1 ± 23.39	**0.044**
Fatigue	39.4 ± 19.90	47.7 ± 29.85	0.215
Pain	10.0 ± 19.04	18.9 ± 27.54	0.157
Dyspnea	13.3 ± 19.94	28.8 ± 27.40	**0.03**
Financial difficulties	15.0 ± 22.88	24.3 ± 23.11	0.15
**Stage IV**			
Physical function	68.3 ± 18.36	50.0 ± 21.68	0.179
Role function	70.8 ± 8.33	47.9 ± 35.00	0.235
Emotional function	77.1 ± 20.83	62.5 ± 14.08	0.177
Cognitive function	70.8 ± 28.46	60.4 ± 25.10	0.53
Social function	75.0 ± 31.92	56.3 ± 34.43	0.385
Global QoL	50.0 ± 18.00	32.3 ± 26.89	0.266
Fatigue	44.4 ± 22.23	61.1 ± 28.49	0.333
Pain	16.67 ± 23.57	41.67 ± 30.86	0.188
Dyspnea	16.7 ± 19.24	50.0 ± 30.86	0.213
Financial difficulties	16.7 ± 19.24	25.0 ± 23.57	0.08

### Independent risk factors for postoperative QoL

The independent risk factors for QoL after gastric cancer surgery were analyzed. Of the several QoL sub-items, the global QoL is the most representative. Therefore, it was used to represent the QoL for the subsequent multivariate regression analysis. The pathological stage (HR 0.13, 95% CI 0.03–0.67, *P* = 0.014), CONUT (HR 3.14, 95% CI 1.01–9.74, *P* = 0.048), and surgical methods (HR 3.13, 95% CI 1.15–8.54, *P* = 0.026) were found to be independent risk factors for general health (Table 13).

**Table 13:** Univariate and multivariate analyses of independent risk factors for postoperative global QoL.

	Univariate analysis			Multivariate analysis		
	HR	95% CI	*P* value	HR	95% CI	*P* value
Age, < 60 versus ≥ 60	1.1	0.43–2.81	0.842			
Sex	1.1	0.43–2.81	0.842			
p Stage, II III IV versus I	0.11	0.23–0.50	**0.005**	0.13	0.03–0.67	**0.014**
CONUT, low versus high	3.51	1.54–7.99	**0.003**	3.14	1.01–9.74	**0.048**
PNI, low versus high	0.4	0.18–0.87	**0.021**	0.98	0.33–2.96	0.974
ALB, low versus high	0.54	0.21–1.21	0.133			
Surgical methods, laparoscopy versus open	3.58	1.42–9.06	**0.007**	3.13	1.15–8.54	**0.026**
Resection, whole versus others	0.86	0.41–1.89	0.734			
CA19-9	0.69	0.32–1.47	0.332			

### Comparison of the values of CONUT score, PNI, and ALB

**Figure 2:**
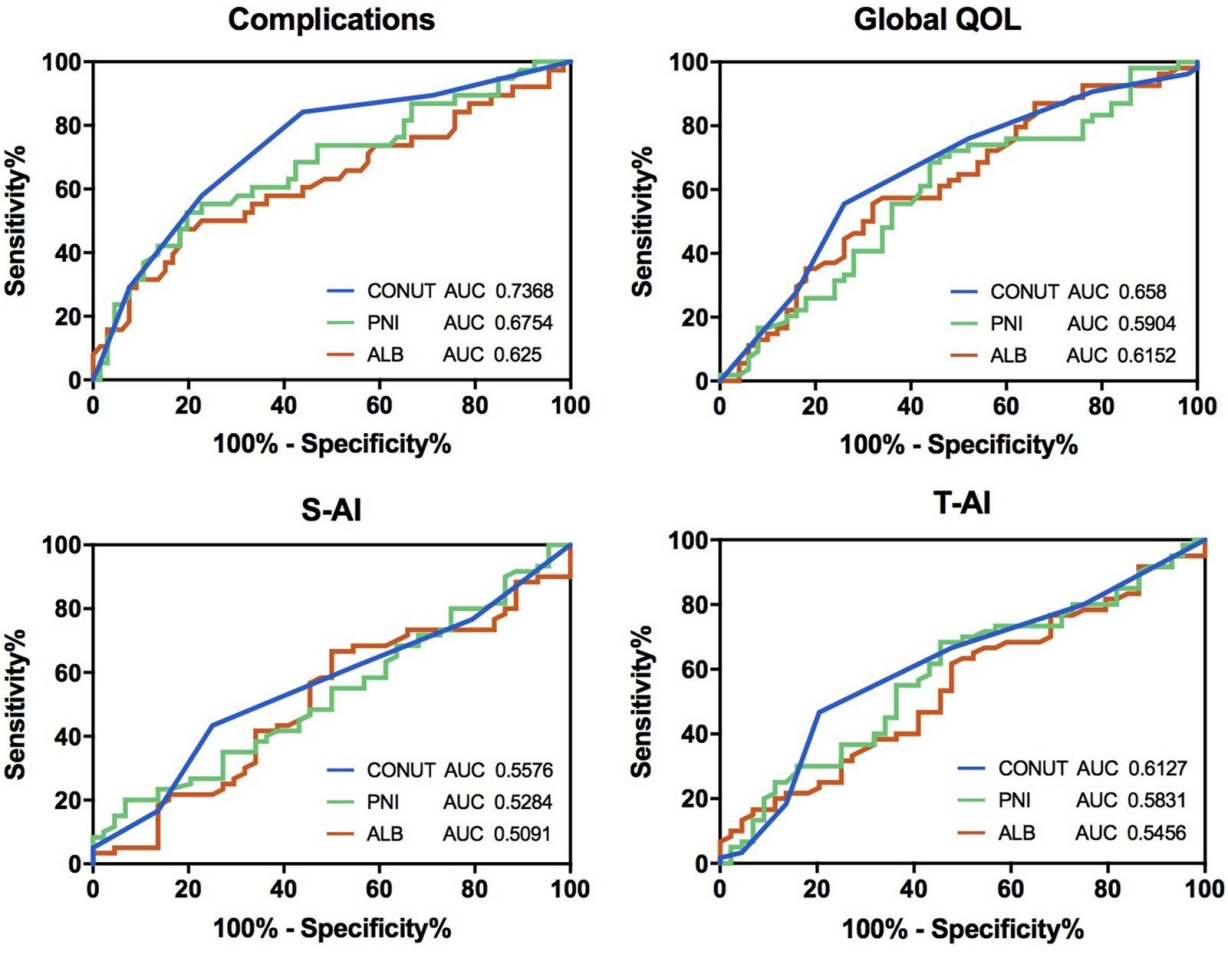
Horizontal comparison of the values of CONUT, PNI, and ALB in complications, global QoL, S-AI and T-AI.

Although the CONUT score was found to be significantly correlated with the postoperative complications, psychological status, and QoL, it must be compared with other nutritional assessment tools. Therefore, the predictive validity of the CONUT score, PNI, and ALB was compared. The CONUT score was demonstrated to be superior to other nutrition assessment methods, especially for the prediction of complications (AUC = 0.7368) (Fig. 2).

#### Associations of CONUT score with OS

Studies have reported^11^ that the CONUT score is closely related to OS of patients with various cancers. Therefore, the univariate and multivariate regression analyses were performed to determine whether the CONUT score is an independent risk factor for OS in patients with gastric cancer after surgery. The univariate analysis exhibited that the postoperative complications (HR 0.22, 95% CI 0.12–0.42, *P* < 0.001), pathological stage (HR 3.69, 95% CI 2.23–6.11, and *P* < 0.001), and S-AI (HR 0.38, 95% CI 0.19–0.76, *P* = 0.006), T-AI (HR 0.43, 95% CI 0.23–0.83, *P* = 0.012), and QoL scores are significantly correlated with OS. And the multivariate analysis exhibited complications (HR 0.43, 95% CI 0.21–0.92, *P* = 0.028), pathological stage (HR 2.26, 95% CI 1.26–4.06, *P* = 0.006), and global QoL scores (HR 15.24, 95% CI 3.22–72.06, *P* = 0.001) as the independent factors for OS (Table 14).

**Table 14:** Results of univariate and multivariate analyses of factors associated with overall survival in gastric cancer patients with operation.

	Univariate analysis			Multivariate analysis		
	HR	95% CI	*P* value	HR	95% CI	*P* value
Sex	1.52	0.76–3.02	0.233			
Age, < 60	1.14	0.56–2.30	0.725			
Hospital stay, < 24 days	1.37	0.76–2.48	0.298			
Postoperative complications	0.22	0.12–0.42	**< 0.001**	0.43	0.21–0.92	**0.028**
CONUT, low versus high	1.66	0.90–3.09	0.107			
PNI, low versus high	0.57	0.31–1.04	0.068			
ALB, low vs. high	0.78	0.43–1.44	0.434			
Stage	3.69	2.23–6.11	**< 0.001**	2.26	1.26–4.06	**0.006**
S-AI, < 36	0.38	0.19–0.76	**0.006**	0.91	0.34–2.46	0.850
T-AI, < 38	0.43	0.23–0.83	**0.012**	2.27	0.93–5.52	0.070
Physical function, < 80	12.09	5.27–27.74	**< 0.001**	1.10	0.30–3.99	0.888
Role function, < 100	6.67	3.19–13.94	**< 0.001**	1.13	0.36–3.54	0.829
Emotional function, < 83.33	4.53	2.38–8.61	**< 0.001**	0.94	0.31–2.84	0.907
Cognitive function, < 83.33	3.28	1.74–6.16	**< 0.001**	1.52	0.56–4.11	0.413
Social function, < 66.67	4.88	2.62–9.10	**< 0.001**	1.53	0.68–3.42	0.307
Global QoL scores, < 66.67	25.20	8.76–72.52	**< 0.001**	15.24	3.22–72.06	**0.001**
Fatigue, < 33.33	0.10	0.03–0.43	**0.002**	0.56	0.11–2.82	0.478

## Discussion

Although several tumor-related nutrition assessment tools have emerged in the past decades, the application of some of these tools is still controversial and criticized in practical clinical work^12^. NRS2002 and other evaluation systems require patients to review body weight changes. However, reviewing the weight changes before onset is difficult for elderly patients as they are usually ignorant of weight changes and may have difficulty remembering. Anthropometric parameters are indispensable in NRS2002 and other classical nutritional evaluation methods. However, these assessment tools have limitations and are ineffective in nutritional screening and assessment of patients with malignant tumors who are extremely weak or edematous as pleural fluid and ascites affect the body weight. Additionally, the sebum thickness of patients with edema cannot accurately reflect the nutritional status; however, it will adversely affect the final nutritional score. Although PG-SGA is an improved nutrition evaluation treatment for patients with cancer, its design cannot accurately reflect the nutritional status of patients with gastric cancer. For example, although breast cancer and stomach cancer are both malignant tumors, the food intake and absorption in patients with gastric cancer is obviously poorer than that in patients with breast cancer. Thus, the same evaluation system would be inaccurate. Additionally, nutrition evaluation systems such as NRS2002 and PG-SGA are too simple to classify malnutrition, and thus are unsuitable for the dynamic evaluation of malnutrition and the effect of nutritional intervention.

Some scholars believe that changes in the internal environment can not only reflect the nutritional status but also help evaluate the risk of postoperative complications^13^. Several subjective parameters are present in clinical nutrition evaluation methods such as NRS2002 that affect the accuracy of the evaluation^14^. The application of CONUT score may open a new door for nutritional assessment and postoperative complication prediction. The CONUT score is an objective evaluation criterion, which is completely based on laboratory parameters. Lymphocyte count is a direct observation index of inflammatory response in patients and also reflects the status of immune surveillance to some extent^15^. ALB can effectively reflect the nutritional status of patients, and it is also related to the liver function reserve and release of inflammatory cytokines from tumor cells^16^. As the main component of the cell membrane, total cholesterol is involved in various signal pathways in tumor pathogenesis and development^17^. Our results also confirm that patients with high CONUT scores are more likely to have postoperative complications, and the finding is consistent with those of other studies^18^. Compared with other nutrition evaluation systems widely used in clinical practice such as NRS2002 and PNI, CONUT score is more accurate and convenient for nutritional evaluation due to the objective and dynamic laboratory results.

The present study exhibited no significant correlation of age, survival status, and length of hospital stay with the CONUT score. This finding is in contrast with that of a study on prognosis of elderly patients with colorectal cancer by Ahiko^18^, which reported that age is significantly correlated with the CONUT score. This difference in research results may be attributed to differences in the subject and age stratification between the two studies. The increasing age results in a gradual decline in body function, leading to poor nutritional status. The role of age in the CONUT score was affected due to the large age span of observers enrolled in our study. Other studies^9,19,20^ have suggested that sex is not associated with the CONUT score. However, our results exhibited that sex is also one of the factors influencing the CONUT score. Differences in the dietary structure and basic metabolic level between men and women in China may affect the nutritional status to a certain extent. CA19-9 is a marker of malignant tumors such as gastrointestinal malignancies and ovarian cancer, and it is also increased in some chronic inflammatory states^21^. Some studies have observed that CA19-9 has a certain correlation with glucose and lipid metabolism^22^. Therefore, the correlation between CA19-9 and CONUT score can be easily understood^23^.

Postoperative complications have received great attention. Some scholars have observed that the CONUT scores of patients with breast cancer, lung cancer, and colorectal cancer are related to postoperative complications^18,24^. Consistent with their findings, we observed a strong correlation between the CONUT score and postoperative grade II complications such as anastomotic leakage. Malnutrition affects the healing and repair of tissues in case of anastomotic leakage. Studies have proved that the incidence of complications such as anastomotic leakage can be greatly reduced by improving the nutritional status of patients^25^. In our study, the CONUT score, PNI, or ALB were strongly associated with postoperative complications, indicating that the nutritional status is a crucial factor affecting the surgical complications. Through univariate and multivariate regression analyses, we observed that the CONUT score is an independent risk factor for postoperative complications, indicating that CONUT score could reflect the relationship between nutritional status and postoperative complications.

The psychological status of patients with cancers has also been receiving considerable attention. Numerous patients with malignant tumors exhibit depression and anxiety, which affects the prognosis of the disease^26^. Studies have exhibited that patients with malnutrition are more likely to develop depression and anxiety^27,28^. Therefore, we used the STAI questionnaire to assess the psychological state of patients with gastric cancer after surgery. Patients with a high preoperative CONUT score also exhibited a high postoperative S-AI score, suggesting that poor preoperative nutrition will increase the anxiety and depression in the short term after surgery. However, the preoperative CONUT score exhibited no significant correlation with postoperative T-AI, suggesting that preoperative nutritional status had little effect on postoperative long-term psychological state. We further performed a logistics regression analysis and observed that the CONUT score was not an independent risk factor for T-AI and S-AI. Our results suggest that nutritional status is not the main factor that affects the postoperative psychological state of patients and that postoperative psychological anxiety may be more affected by other factors.

Postoperative QoL has also been studied in recent years, and a close correlation of the nutritional status with the QoL has been observed^29^. Therefore, we evaluated the postoperative QoL in patients with gastric cancer by using EORTC QLQ-C30. The EORTC QLQ-C30 assessed the QoL of patients in terms of function, symptoms, and overall health^28^ (Global QoL). Patients with a high CONUT score exhibited lower scores for physical function, role function, cognitive function, emotional function, social function, and global QoL than patients with a low CONUT score. Additionally, patients with high CONUT scores exhibited higher scores for fatigue, pain, dyspnea, and financial difficulty than patients with low CONUT scores. According to the scoring criteria of the EORTC QLQ-C30 scale, high scores for the function and global QoL indicate better functional status and life quality, whereas high scores for the symptom area indicate more symptoms or problems (a lower QoL). It also suggests that patients with a high CONUT score are associated with poor QoL. We conducted univariate and multivariate regression analyses and observed that the CONUT score is an independent risk factor for the QoL of patients with gastric cancer after surgery. In addition, we evaluated the relationship between COUNT score and preoperative QoL in patients with gastric cancer. The symptoms of the digestive system are closely related to the CONUT score, but there is no obvious correlation in other aspects. We found that the results of QoL score before surgery were significantly worse than after surgery. Confusion and pain caused by the disease were far more influential than the nutritional status. Preoperative QoL may be more influenced by the disease itself, while nutrition did not dominate. Several studies have observed that the CONUT score is associated with postoperative OS of patients^30^. Therefore, we also analyzed the relationship between CONUT score and postoperative OS of patients with gastric cancer. The CONUT score exhibited no significant correlation with OS and could not be used to predict the prognosis of patients. This finding is concurrent with that of other studies^31^. Predicting the prognosis of patients undergoing gastric cancer surgery is challenging^32^. The postoperative TNM stage is a decisive factor affecting the prognosis of patients with gastric cancer. Additionally, postoperative complications, accidents, and economic status may affect OS of these patients. Although nutritional status influences postoperative OS, it is not a major determinant.

The present study has certain limitations. The single-center retrospective design of the study may introduce a certain degree of data bias. However, we screened cases strictly and ensured the validity and representativeness of the data as much as possible in the statistical process. Moreover, we attempted to make the questionnaire as detailed as possible in the follow-up process to reduce the loss of effective data. With the increasing geriatric population, the nutritional status of elderly patients has attracted attention. Future studies in elderly patients with a larger sample size would allow the further analysis of the application of the CONUT score.

## Conclusion

The CONUT score is a simple and objective measure that can reduce the workload of clinicians. Therefore, it has a certain clinical value in preoperative nutrition assessment of gastric cancer and can be used to predict postoperative complications and QoL.
